# Assessing interstitial fluid dynamics in type 2 diabetes mellitus and prediabetes cases through diffusion tensor imaging analysis along the perivascular space

**DOI:** 10.3389/fnagi.2024.1362457

**Published:** 2024-03-07

**Authors:** Rukeye Tuerxun, Koji Kamagata, Yuya Saito, Christina Andica, Kaito Takabayashi, Wataru Uchida, Seina Yoshida, Junko Kikuta, Hiroki Tabata, Hitoshi Naito, Yuki Someya, Hideyoshi Kaga, Mari Miyata, Toshiaki Akashi, Akihiko Wada, Toshiaki Taoka, Shinji Naganawa, Yoshifumi Tamura, Hirotaka Watada, Ryuzo Kawamori, Shigeki Aoki

**Affiliations:** ^1^Department of Radiology, Juntendo University Graduate School of Medicine, Tokyo, Japan; ^2^Faculty of Health Data Science, Juntendo University, Chiba, Japan; ^3^Department of Radiological Sciences, Graduate School of Human Health Sciences, Tokyo Metropolitan University, Tokyo, Japan; ^4^Sportology Center, Juntendo University Graduate School of Medicine, Tokyo, Japan; ^5^Department of Metabolism and Endocrinology, Juntendo University Graduate School of Medicine, Tokyo, Japan; ^6^Graduate School of Health and Sports Science, Juntendo University, Chiba, Japan; ^7^Department of Functional Brain Imaging, National Institutes for Quantum and Radiological Science and Technology, Chiba, Japan; ^8^Department of Innovative Biomedical Visualization, Nagoya University Graduate School of Medicine, Nagoya, Japan; ^9^Department of Radiology, Nagoya University Graduate School of Medicine, Nagoya, Japan

**Keywords:** glymphatic system, insulin resistance, type 2 diabetes mellitus, prediabetes, diffusion-magnetic resonance imaging

## Abstract

**Background and purpose:**

Glymphatic system in type 2 diabetes mellitus (T2DM) but not in the prodrome, prediabetes (Pre-DM) was investigated using diffusion tensor image analysis along the perivascular space (DTI-ALPS). Association between glymphatic system and insulin resistance of prominent characteristic in T2DM and Pre-DM between is yet elucidated. Therefore, this study delves into the interstitial fluid dynamics using the DTI-ALPS in both Pre-DM and T2DM and association with insulin resistance.

**Materials and methods:**

In our cross-sectional study, we assessed 70 elderly individuals from the Bunkyo Health Study, which included 22 with Pre-DM, 18 with T2DM, and 33 healthy controls with normal glucose metabolism (NGM). We utilized the general linear model (GLM) to evaluate the ALPS index based on DTI-ALPS across these groups, considering variables like sex, age, intracranial volume, years of education, anamnesis of hypertension and hyperlipidemia, and the total Fazekas scale. Furthermore, we have explored the relationship between the ALPS index and insulin resistance, as measured by the homeostasis model assessment of insulin resistance (HOMA-IR) using GLM and the same set of covariates.

**Results:**

In the T2DM group, the ALPS index demonstrated a reduction compared with the NGM group [family-wise error (FWE)-corrected *p* < 0.001; Cohen's *d* = −1.32]. Similarly, the Pre-DM group had a lower ALPS index than the NGM group (FWE-corrected *p* < 0.001; Cohen's *d* = −1.04). However, there was no significant disparity between the T2DM and Pre-DM groups (FWE-corrected *p* = 1.00; Cohen's *d* = −0.63). A negative correlation was observed between the ALPS index and HOMA-IR in the combined T2DM and Pre-DM groups (partial correlation coefficient *r* = −0.35, *p* < 0.005).

**Conclusion:**

The ALPS index significantly decreased in both the pre-DM and T2DM groups and showed a correlated with insulin resistance. This indicated that changes in interstitial fluid dynamics are associated with insulin resistance.

## 1 Introduction

Characteristics of type 2 diabetes mellitus (T2DM), such as insulin resistance, are known to lead to nerve cell damage and neuroinflammation (Strittmatter et al., 1993; Kamiyama et al., 2010; Iliff et al., 2012; Arnold et al., 2018; Kim et al., 2018), which are highly recognized as contributing factors to neurological disorders like stroke and Alzheimer's disease (AD; Giorda et al., 2007; Janghorbani et al., 2007). Previous research has shown that T2DM is related to the glymphatic system within the central nervous system (CNS; Jiang et al., 2017). In recent years, the hypothesis for lymphatic system has emerged, describing brain's waste clearance mechanism involving cerebrospinal fluid (CSF) flow. This system function akin to brain's inherent self-cleaning mechanism, actively eliminating brain waste and harmful substances, thereby playing a pivotal role in sustaining brain health. According to this hypothesis, with arterial pulsations and respiratory movements, CSF flows from the subarachnoid space into the perivascular space that encircles the artery. Subsequently, the CSF diffuses into the cerebral tissue through water channels known as aquaporin-4 (AQP4) located in the terminal extensions of astrocytic cells (Iliff and Nedergaard, 2013). Consequently, CSF intermixes with the intrinsic waste solutes and interstitial fluid (ISF) within the cerebral tissue, and ultimately, effluxes into the perivascular space that encircles the vein, leading to its influx into the peripheral lymphatic system (Iliff et al., 2012). The system assists in clearing soluble proteins, like amyloid-β (Aβ), and metabolic products. It also contributes to the distribution of glucose, lipids, amino acids, and neuromodulators (Iliff et al., 2012, 2013; Jessen et al., 2015). A key component of the glymphatic system, AQP4 is pivotal in improving the flow of CSF and maintains waste clearance within the brain parenchyma (Benveniste et al., 2019). AQP4 is also decreased in T2DM, leading to the impairment of glymphatic system (Zhang et al., 2016; Ward et al., 2019). Thus, the glymphatic system dysfunction in T2DM might induce Aβ accumulation.

Presently, the benchmark to evaluate glymphatic system function is intrathecal injection of gadolinium- based contrast agents (GBCA; Taoka and Naganawa, 2020), and a previous rat study using contrast-enhanced magnetic resonance imaging (MRI) with intrathecal GBCA demonstrated that the glymphatic system might be lower in rat with T2DM than in rat with a normal glucose metabolism (NGM; Jiang et al., 2017). However, intrathecal GBCA injections may cause anaphylactic reactions and neurotoxic effects (Li et al., 2008; Edeklev et al., 2019), and they are invasive for humans. Therefore, it is challenging to utilize intrathecal GBCA for assessing the glymphatic function in humans. In response to existing challenges, the ALPS index, related with the perivascular space and employing diffusion tensor image analysis (DTI-ALPS) through diffusion MRI (dMRI), has emerged as a cutting-edge non-invasive MRI technique. It focuses is to monitor the glymphatic system's functionality, via the dynamics of CSF/ISF (Taoka et al., 2017a; Taoka and Naganawa, 2021). The DTI-ALPS method yields the ALPS index, a measure that compares the diffusion capacity parallel to the perivascular space surrounding the deep medullary vein at the lateral ventricle body with the diffusion capacity perpendicular to major fiber tracts (Taoka et al., 2017a). Given its strong correlation with the glymphatic clearance function, observed using MRI with intrathecal GBCA injection (the current gold standard method), the ALPS index is being viewed as a potential pivotal biomarker for evaluating the glymphatic system's efficiency (Taoka et al., 2017a, 2022; Zhang et al., 2021; Saito et al., 2023a,b,c,d; Tatekawa et al., 2023).

A human study using the DTI-ALPS suggested that patients with T2DM could have a lower glymphatic system function than individuals with NGM (Yang et al., 2020). As of now, we have not encountered any studies that delve into alterations in the glymphatic system using the ALPS index during the prodromal stage of T2DM, known as prediabetes (Pre-DM). Furthermore, the specific factor leading to diminished glymphatic system in patients with T2DM remains elusive. Although the insulin resistance could reportedly cause the Aβ degrading delays and the accumulation of Aβ in T2DM cases, as compared to that seen in NGM cases (Ho et al., 2004; Yang et al., 2013; Mehla et al., 2014; Vandal et al., 2014), at present, there is no documented link between the glymphatic system and insulin resistance.

Therefore, we hypothesized that the ISF dynamics in patients with Pre-DM declined before they developed T2DM, as compared to participants with NGM, and the risk factor is the level of insulin resistance. This research sought to assess the ALPS index across the T2DM, Pre-DM, and NGM groups. Additionally, we aim to elucidate the relationship between insulin resistance level and the ALPS index.

## 2 Materials and methods

### 2.1 Participants

Our research was sanctioned by the Institutional Review Board, and prior to the assessment, all participants furnished written informed consent. This study included 70 older adults residing in the community (37 male and 33 female; mean age, 71.4 years) recruited from the Bunkyo Healthy Study (Someya et al., 2019; Table 1). This cohort comprised 18 participants with T2DM, 22 participants with prediabetes (Pre-DM), and 30 with NGM. In the T2DM group, 11 individuals took antidiabetic medications to manage their blood sugar levels. The patients with T2DM in this study were diagnosed by using 2019 Japanese Clinical Practice Guideline for Diabetes by The Japan Diabetes Society (Table 2; Seino et al., 2010). In this study, participants were categorized into T2DM, Pre-DM, and NGM based on fasting plasma glucose (FPG), 75 g oral glucose tolerance test (2-h OGTT), and glycated hemoglobin (HbA1c) levels. To diagnose T2DM, patients must have an FPG ≥ 126 mg/dL, or a 2-h OGTT ≥ 200 mg/dL, and an HbA1c ≥ 6.1%. Pre-DM is indicated by an HbA1c level between 5.6 and 6.0%, an FPG of 110–125 mg/dL, or a 2-h OGTT of 140–199 mg/dL. NGM requires simultaneously meeting all these criteria: FPG < 110 mg/dL, 2-h OGTT < 140 mg/dL, and HbA1c < 5.4%. These standards align with widely accepted clinical guidelines for diabetes classification. The participants excluded from this study were individuals with brain tumor or cardiovascular disease, those who had suffered significant traumatic brain injuries, or those diagnosed with mental or neural disorders. To investigate the association between the ALPS index and insulin resistance, we sourced the homeostasis model assessment for insulin resistance (HOMA-IR) data directly from the Bunkyo Health Study database (Matthews et al., 1985). Additionally, we collected the cognitive function data, which was evaluated using the Montreal Cognitive Assessment-Japanese version (MoCA-J). Additionally, white matter hyperintensities were assessed with the total Fazekas scale, summing scores for periventricular and subcortical areas.

**Table 1:** Clinical and demographic profile of the research cohort.

	**NGM**	**Pre-DM**	**T2DM**	***p*** **-value**		
				**NGM vs. Pre-DM**	**NGM vs. T2DM**	**Pre-DM vs. T2DM**
*N*	30	22	18	–		
Age, year	70.3 ± 4.8	72.1 ± 4.0	72.6 ± 6.0	0.092	0.145	0.902
Sex (female/male)	16/14	12/10	5/13	0.931	0.084	0.088
Education years, year	14.3 ± 2.2	14.1 ± 1.9	13.8 ± 2.2	0.840	0.601	0.755
BMI	21.6 ± 3.0	23.1 ± 2.7	24.1 ± 2.9	0.139	0.016^*^	0.344
Hypertension/non-hypertension	15/15	15/7	12/6	0.190	0.260	0.919
Hyperlipidemia/non-hyperlipidemia	14/16	18/4	13/5	0.01^*^	0.084	0.470
MoCA-J	26.2 ± 2.5	25.5 ± 2.7	25.8 ± 2.6	0.383	0.556	0.828
HOMA-IR	0.8 ± 0.5	1.2 ± 0.4	1.7 ± 1.3	0.007^*^	< 0.001^***^	0.469
Fasting plasma glucose, mg/dL	92.4 ± 5.7	99.0 ± 7.8	121.0 ± 20.3	0.021^*^	< 0.001^***^	0.003^*^
2-h OGTT, mg/dL	105.0 ±21.4	154.0 ± 21.0	254.4 ± 64.5	< 0.001^***^	< 0.001^***^	0.004^*^
HbA1c, %	5.5 ± 0.3	5.9 ± 0.2	6.6 ± 0.9	< 0.001^***^	< 0.001^***^	0.026^*^
T-cho, mg/dL	208.0 ± 31.1	221.5 ± 41.0	194.4 ± 41.8	0.404	0.175	0.044^*^
HDL, mg/dL	67.6 ± 12.5	61.6 ± 16.3	62.1 ± 20.7	0.074	0.068	0.897
LDL, mg/dL	121.2 ± 30.0	141.8 ± 34.5	111.0 ± 32.3	0.054	0.317	0.008^*^
Total Fazekas scale	2.1 ± 0.61	2.5 ± 0.8	2.78 ± 1.0	0.212	0.004^*^	0.368
Periventricular WM, *N* (0/1/2/3)	1/28/1/0	0/19/3/0	1/10/7/0	0.299	0.001^*^	0.046^*^
Deep WM, *N* (0/1/2/3)	1/25/4/0	0/15/6/1	0/11/6/1	0.241	0.109	0.865

±, standard deviation; N, represents the number of participants in this project; OGTT, oral glucose tolerance test; HbA1c, hemoglobin A1c; T-cho, total cholesterol; HDL, high-density lipoproteins; LDL, low-density lipoprotein; HOMA-IR, Homeostatic Model Assessment for Insulin Resistance; MoCA-J, Japanese version of Montreal Cognitive Assessment.

^*^p < 0.05; ^***^p < 0.001.

**Table 2:** Classification criteria for type 2 diabetes mellitus and prediabetes (based on the 2019 Japanese Clinical Practice Guideline for Diabetes by The Japan Diabetes Society).

	**T2DM**	**Pre-DM**	**NGM**
Fasting Plasma Glucose, mg/dL	≥126	110–125	< 110
2-h OGTT, mg/dL	≥200	140–199	< 140
HbA1c, %	≥6.1	5.6–6.0	< 5.4

OGTT, oral glucose tolerance test; HbA1c, hemoglobin A1c; JDS, Japan Diabetes Society.

### 2.2 MRI acquisition

To enhance the precision of our data, we employed the MAGNETOM Prisma 3T MRI scanner (Siemens, Erlangen, Germany) with a 64-channel head coil to collect the entire brain dMRI dataset. The technique used was echo planar imaging with multiple slices in the anterior–posterior axis, featuring 64 diffusion gradient directions and having a *b*-value of 1,000 s/mm^2^. Furthermore, a non-diffusion-weighted volume (*b* = 0 s/mm^2^) was also recorded with these settings: TR/TE = 3,300/70 ms, FOV = 229 mm × 229 mm, matrix size = 130 × 130, 1.8 mm isotropic voxels, and acquisition time = 7 min and 29 s. Furthermore, 3D MP-RAGE T1-weighted images were also acquired with the following parameters: TR/TE, 2,300/2.32 ms; TE, 900 ms; FOV, 240 × 240 mm; matrix size, 256 × 256; resolution, 0.9 × 0.9 mm; slice thickness, 0.9 mm, and acquisition time, 6 min and 25 s. Moreover, to correct for the magnetic susceptibility-induced distortions associated echo planar imaging acquisition, we obtained both standard and reverse phase-encoding image without diffusion weighting, commonly referred to as blip-up and blip-down sequences (Andersson and Sotiropoulos, 2016).

### 2.3 MRI processing

We conducted processing of the dMRI data utilizing FSL (https://fsl.fmrib.ox.ac.uk/fsl/fslwiki) and MRTrix3 (https://www.mrtrix.org), as described in recent studies outlining optimal preprocessing procedures (Tournier et al., 2019). Initially, MR magnitude images underwent denoising via the Marchenko–Pastur principal component analysis (MP-PCA; Cordero-Grande et al., 2019) and had Gibbs artifacts rectified (Kellner et al., 2016). Subsequently, to mitigate potential biases from the Rician noise distribution, we harnessed an analytical strategy, using the noise standard deviation gauged through MP-PCA. Furthermore, we utilized the “EDDY” and “TOPUP” commands from the FSL suite to address susceptibility-induced geometric distortions, eddy current-induced distortions, and participant head motion in the dMRI data. These comprehensive steps were critical in mitigating susceptibility and geometric distortions, thereby significantly reducing potential biases in our analysis (Andersson and Sotiropoulos, 2016; Graham et al., 2017). The refined dMRI images were then aligned to the DTI model, leveraging an estimation via ordinary least squares, producing diffusion coefficient maps with the x-axis (right–left; Dxx), y-axis (anterior–posterior; Dyy), and z-axis (inferior–superior; Dzz) and FA maps using FSL's “dtifit” function (Jenkinson et al., 2012). Lastly, we reviewed FA maps and the diffusion coefficient maps to confirm the absence of significant artifacts like pronounced geometric distortion, signal losses, or extensive movement.

### 2.4 ALPS index calculation

**Figure 1:**
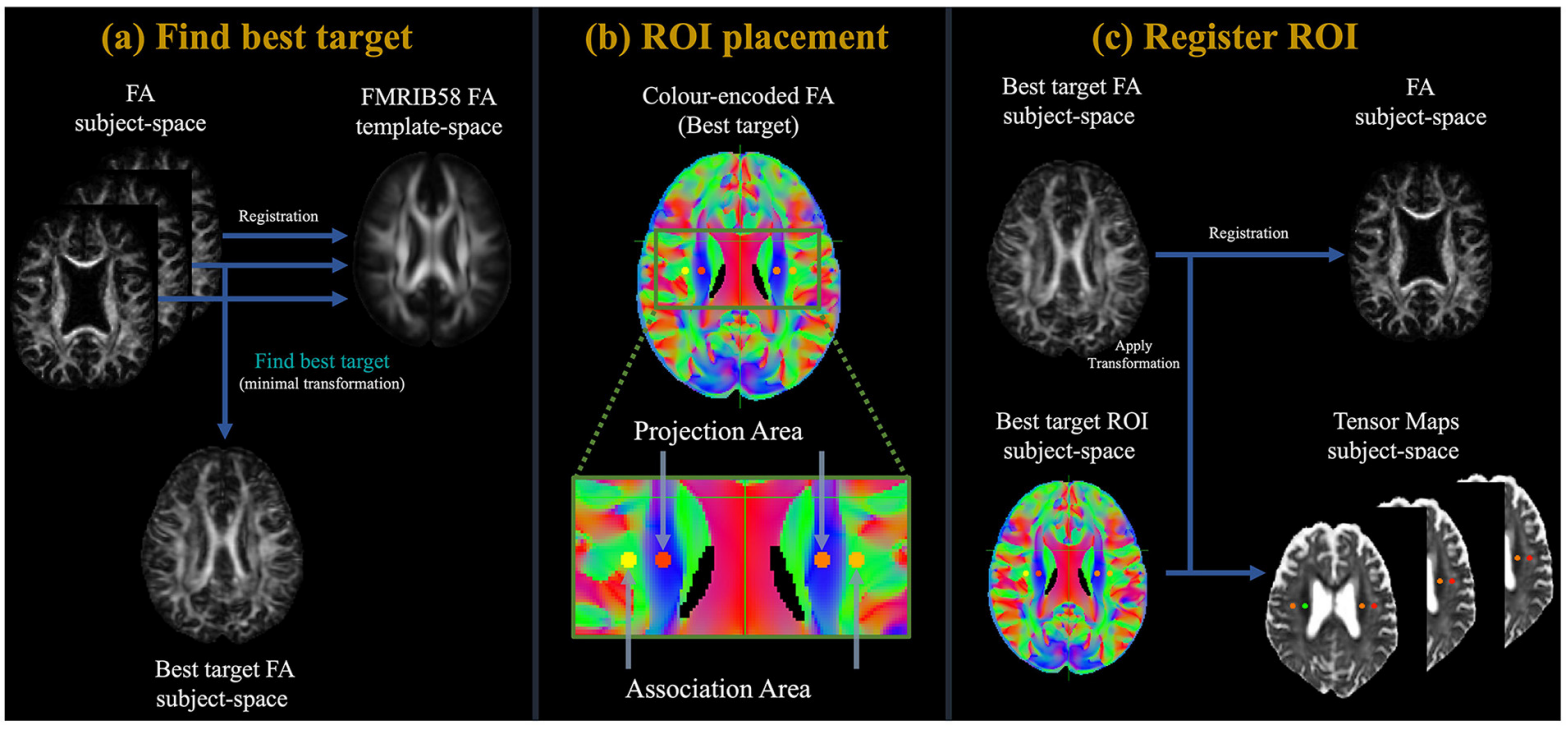
Flowchart of the study process. **(a)** Initially, the FA maps of all participants underwent a linear followed by non-linearly registration to the high-definition FMRIB58_FA standard-space reference image. Subsequently, based on the criterion of minimal deformation, specifically, the lowest sum of squared discrepancies, we have selected the appropriate participant to determine the region-of-interest (ROI). **(b)** Employing the native color-coded FA map from the chosen participant, we set ROIs measuring 5 mm in diameter next to the lateral ventricle bodies within both projection and association regions across both hemispheres. In the projection region, the domain fibers are oriented along the z-axis, standing perpendicular to the x- and y-axes. Conversely, in the association region, principal fibers are oriented along the y-axis, perpendicular to the x- and z-axes. **(c)** After this, the delineated ROIs underwent registration with an individual FA map. The ALPS index was then derived using Equation 1.

The ALPS index, originating from DTI-ALPS, is primarily calculated based on the method illustrated in Figure 1 (Taoka et al., 2017a, 2022; Kamagata et al., 2022; Tang et al., 2022). Initially, the FA maps from all participants underwent linear registration, followed by non-linear alignment to the high-definition FMRIB58_FA standard-space reference image. Subsequently, based on the criterion of minimal deformation, specifically, the lowest sum of squared discrepancies, we have selected the appropriate participant to determine the region-of-interest (ROI). Employing the native color-coded FA map from the chosen participant, we set ROIs measuring 5.4 mm in diameter next to the lateral ventricle bodies within both projection and association regions across both hemispheres. In the projection region, the principal fibers were oriented along the z-axis, orthogonal to the x- and y-axes. Conversely, in the association region, the principal fibers were aligned along the y-axis, orthogonal to the x- and z-axes. Subsequently, the identified ROIs were aligned to an individual's FA map. Manually verified the placement of the ROIs for each participant. As all ROIs were accurately positioned, no manual adjustments were required. To sum up, the ALPS index was formulated in the following formula:


(1) $$\begin{array}{c} ALPS-index=\frac{mean\left(D_{xxproj},D_{xxassoc}\right)}{mean\left(D_{yyproj},D_{zzassoc}\right)} \end{array}$$

The provided metric represents the average of the x-axis diffusivity in the projection region (*D*_*xxproj*_) over the x-axis diffusivity in the association region (*D*_*xxassoc*_) relative to the average y-axis diffusivity in the projection region (*D*_*yyproj*_) and z-axis diffusivity in the association region (*D*_*zzassoc*_). When the ALPS index approaches 1.0, it suggests minimal diffusion along the perivascular space, whereas a value exceeding 1.0 signifies increased diffusivity.

### 2.5 Statistical analysis

All data analyses were done with IBM SPSS Statistics for Windows, version 22.0 (IBM Corporation, Armonk, NY, USA). To contrast participant characteristics, Fisher's exact test was applied for categorical data and the Kruskal–Wallis test for continuous data. By employing GLM, we examined the mean ALPS index from the left to the right hemispheres, across the T2DM, Pre-DM and NGM groups. This method controlled for various factors, including years of education, sex, age, intracranial volume (ICV) estimated from T1-weighted brain scans via FreeSurfer (http://surfer.nmr.mgh.harvard.edu), anamnesis of hypertension and hyperlipidemia, and the total Fazekas scale as covariates. To ensure the accuracy of our model, multicollinearity among these covariates was checked first by examining the variance inflation factor (VIF) for each. The results showed that all variables had VIF values of < 5, demonstrating that multicollinearity did not significantly compromise the integrity of our statistical model. Furthermore, we inspected each study participant's images and did not detect white matter lesions within the ROI. To determine the effect magnitude between the three groups, we computed Cohen's *d* as described (Equations 2 and 3):


(2) $$\begin{array}{c} S_{c}=\sqrt{\frac{n_{1}s_{1}^{2}\;+\;n_{2}s_{2}^{2}}{n_{1}\;+\;n_{2}}} \end{array}$$

(3) $$\begin{array}{c} Cohen^{\prime }s\;d=\frac{{\bar{M}}_{2}-{\bar{M}}_{1}}{S_{c}} \end{array}$$

Here, *n*_1_ and *n*_2_ denote the participant counts in populations 1 and 2. ${\bar{M}}_{1}$ and ${\bar{M}}_{2}\;$ stand for the mean of the variables in these populations, whereas *s*_1_ and *s*_2_ are their respective standard deviations. For this research, we determined Cohen's *d* for the groups T2DM, Pre-DM, and NGM. As the site impact diminishes, the Cohen's *d* value tends toward a smaller figure. In the absence of a site effect, the value of Cohen's *d* should be zero. In this research, following to Cohen's recommendations (Hopkins et al., 2009), value of 0.2 < |*d*| ≤ 0.5, 0.5 < |*d*| ≤ 0.8, and 0.8 < |*d*| were categorized as small, medium, and large impacts, respectively.

Moreover, to evaluate the relation of the ALPS index with insulin resistance in the Pre-DM and T2DM groups, the significance relation of the ALPS index with HOMA-IR was confirmed using a GLM with sex, age, ICV, years of education, anamnesis of hypertension and hyperlipidemia, and the total Fazekas scale (the sum of the Fazekas scales for periventricular and subcortical areas) as covariates. The partial correlation coefficient (*r*) was calculated as the effect size of correlation in the GLM. For all statistical evaluations, a *p*-value < 0.05 was deemed to indicate statistically significant. In the group comparison, *p*-values were corrected by the family-wise error (FWE) correction.

## 3 Results

### 3.1 Clinical and demographic profile of the research cohort

Table 1 provides a detailed overview of the clinical and demographic features of our research participants. Following the group analysis through the application of Fisher's exact and Kruskal–Wallis testing methods, we found that factors such as sex, age, years of education, MoCA-J, and history of hypertension did not exhibit any notable differences between the NGM, Pre-DM, and T2DM groups (*p* > 0.05). However, the were distinct variations in history of hyperlipidemia across the three groups, along with a significant difference in the total Fazekas scale between the T2DM and NGM groups (*p* < 0.05).

### 3.2 Group differences

**Figure 2:**
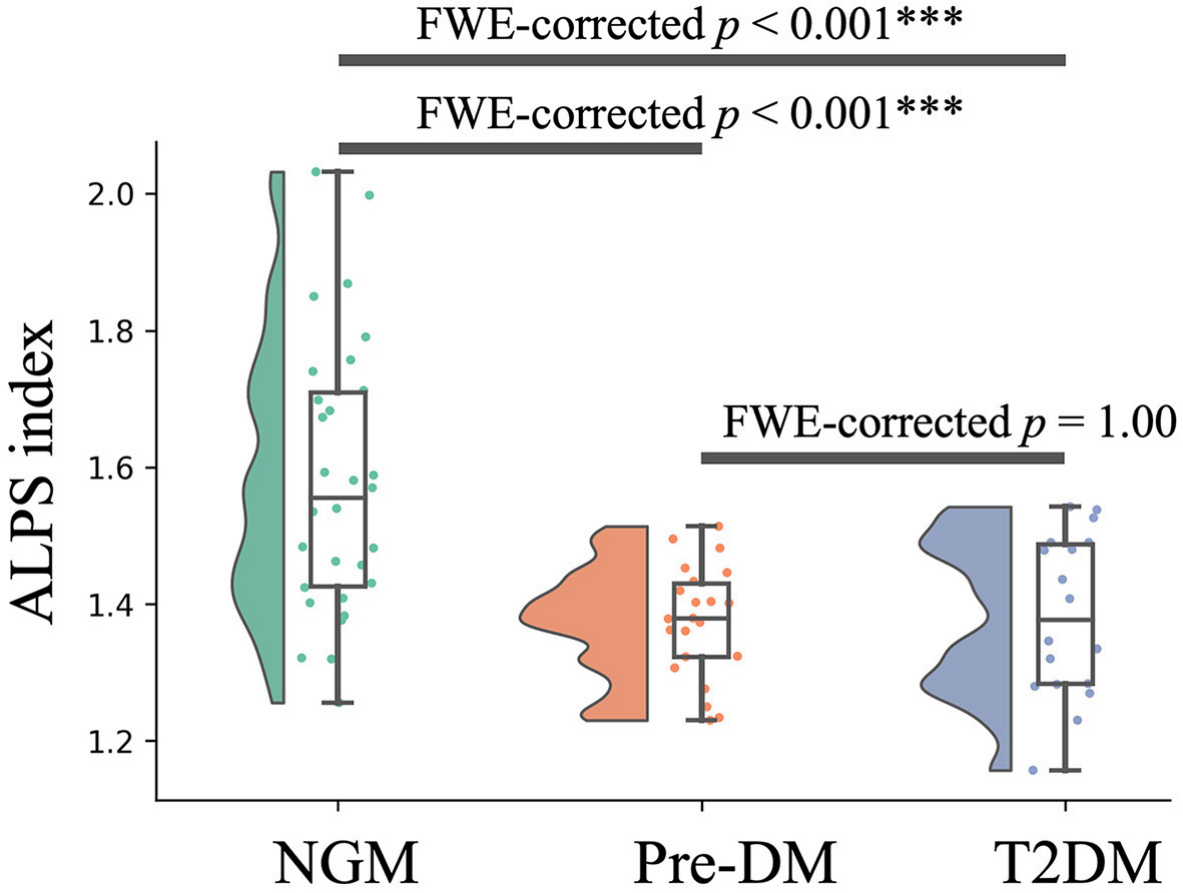
Variations in the ALPS index across groups. The y-axis displays the ALPS index, whereas the x-axis categorizes the groups. Boxplots illustrate the variation in the ALPS index across the NGM (green), Pre-DM (red), and T2DM (blue) groups. The boxplots illustrate the interquartile range of the dataset, indicating the highest and lowest values with the extension of whiskers, while covering 50% of individual participant values. The ALPS index was compared among the T2DM, Pre-DM, and NGM groups through a GLM analysis with the covariates, such as sex, years of education, age, ICV, the anamnesis of hypertension and hyperlipidemia, and the total Fazekas scale. NGM, normal glucose metabolism; Pre-DM, prediabetes; T2DM, Type 2 diabetes mellitus, GLM, general linear model; ICV, intracranial volume; ^***^p < 0.001.

As depicted in Figure 2 and Table 3, variations in the ALPS index across the groups are evident. The T2DM group had a notably reduced ALPS index compared to the NGM group [FWE-corrected *p* < 0.001; Cohen's *d* = −1.32 [${\bar{M}}_{1}$, NGM; ${\bar{M}}_{2}$, T2DM]]. In a similar vein, the Pre-DM group's ALPS index was significantly diminished relative to the NGM group [FWE-corrected *p* < 0.001; Cohen's *d* = −1.04 [${\bar{M}}_{1}$, NGM; ${\bar{M}}_{2}$, Pre-DM]]. Despite a subtle decrease in the ALPS index for the T2DM group compared to Pre-DM group, the difference wasn't statistically significant (FWE-corrected *p* = 1.00), it was marginally lower in the T2DM group relative to the Pre-DM group [Cohen's *d* = −0.63 [${\bar{M}}_{1}$, Pre-DM; ${\bar{M}}_{2}$, T2DM]]. This pattern indicates a progressive decrease in the ALPS index across the three groups, in descending order: NGM > Pre-DM > T2DM groups (Figure 2 and Table 3).

**Table 3:** Group comparison of the ALPS index.

	**Mean** ±**SD**			**Cohen's** ***d***		
	**NGM**	**Pre-DM**	**T2DM**	**NGM vs. Pre-DM**	**NGM vs. T2DM**	**Pre-DM vs. T2DM**
ALPS index	1.58 ± 0.20	1.43 ± 0.08	1.36 ± 0.14	−1.04	−1.32	−0.63

NGM, normal glucose metabolism; Pre-DM, prediabetes; T2DM, Type 2 diabetes mellitus.

### 3.3 Correlation analyses

**Figure 3:**
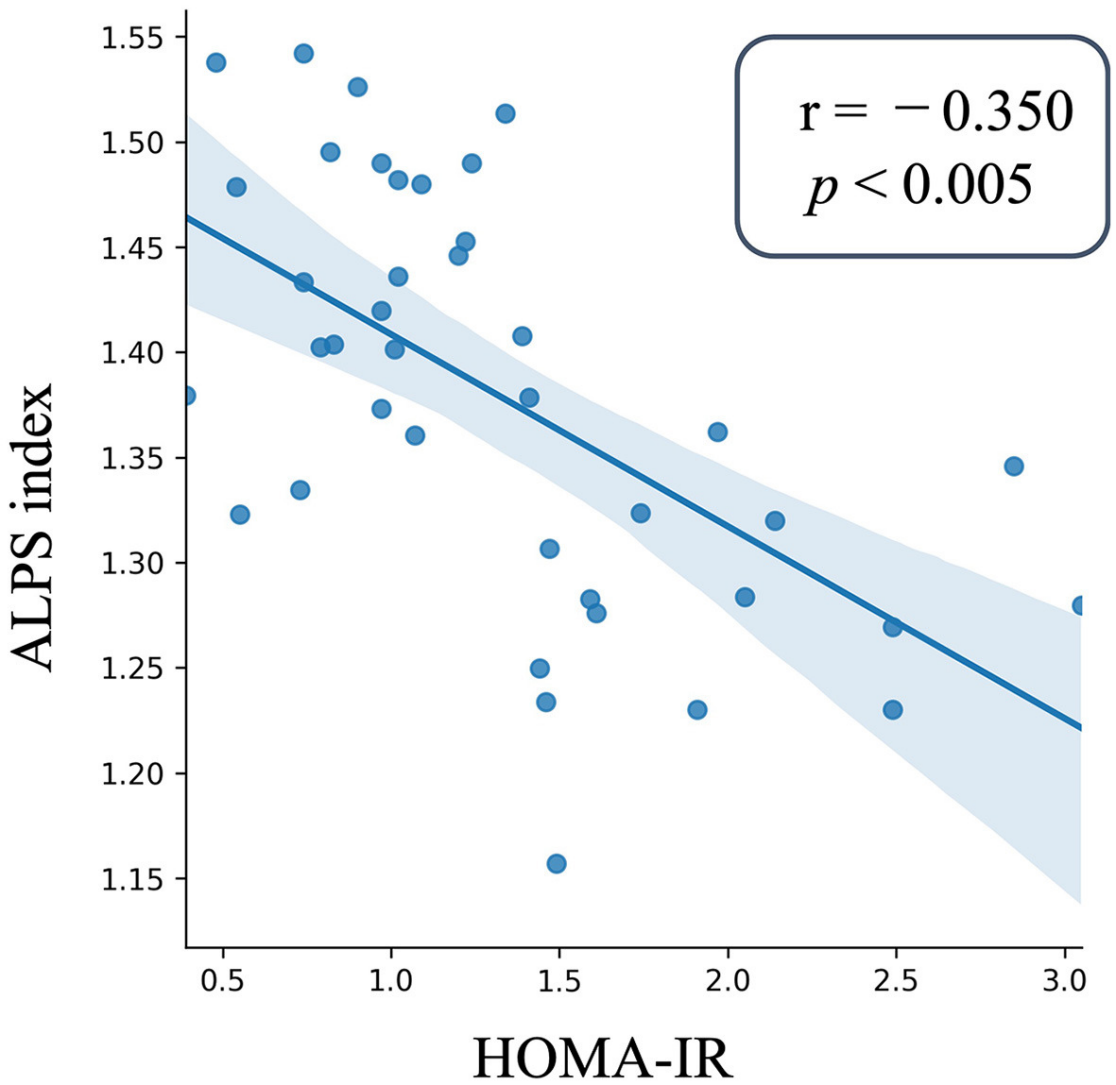
Scatter plot of the ALPS index and insulin resistance. The ALPS index is plotted on the y-axis, whereas the x-axis presents the HOMA-IR values for those with Pre-DM and T2DM. The depicted blue line is the outcome of a linear regression, and the surrounding error bars represent the 95% confidence interval. The degree and significance of the relationship, denoted by the partial correlation coefficient (*r*) and *p*-value, were derived using GLM. The ALPS index as the independent factor was analyzed against covariates like age, sex, years of education, ICV, the anamnesis of hypertension and hyperlipidemia, and the total Fazekas scale. The ALPS index showed a significant negative correlation with HOMA-IR in the patients with Pre-DM and T2DM. NGM, normal glucose metabolism; Pre-DM, prediabetes; T2DM, Type 2 diabetes mellitus, GLM, general linear model; ICV, intracranial volume; HOMA-IR, Homeostasis Model Assessment of Insulin Resistance.

To ascertain the relationship between insulin resistance and the ALPS index within the Pre-DM and T2DM groups, we examined the significance of the ALPS index in relation to HOMA-IR. This examination was facilitated by a GLM, considering factors like sex, years of education, age, ICV, anamnesis of hypertension and hyperlipidemia, and the total Fazekas scale. The analysis revealed a distinct inverse correlation between HOMA-IR and the ALPS index for the merged T2DM and Pre-DM groups (partial correlation coefficient *r* = – 0.35, *p* < 0.005, Figure 3).

## 4 Discussion

This research investigated whether the interstitial fluid dynamics deteriorates in individuals with Pre-DM before progressing to T2DM, compared to those with NGM. Additionally, the correlation between the ALPS index and the insulin resistance level was analyzed. Consequently, the ALPS index for both Pre-DM and T2DM groups serves as a potential measure for assessing interstitial fluid dynamics, showing a noticeable reduction when contrasted with the NGM group. This result might imply that the ISF dynamics in individuals with Pre-DM declined before becoming patients with T2DM compared with participants with NGM. Moreover, a considerable negative correlation was established between the ALPS index and HOMA-IR related to the severity of insulin resistance in the combined T2DM and Pre-DM groups, this finding means that the level of insulin resistance increases as the ALPS index decreases.

**Figure 4:**
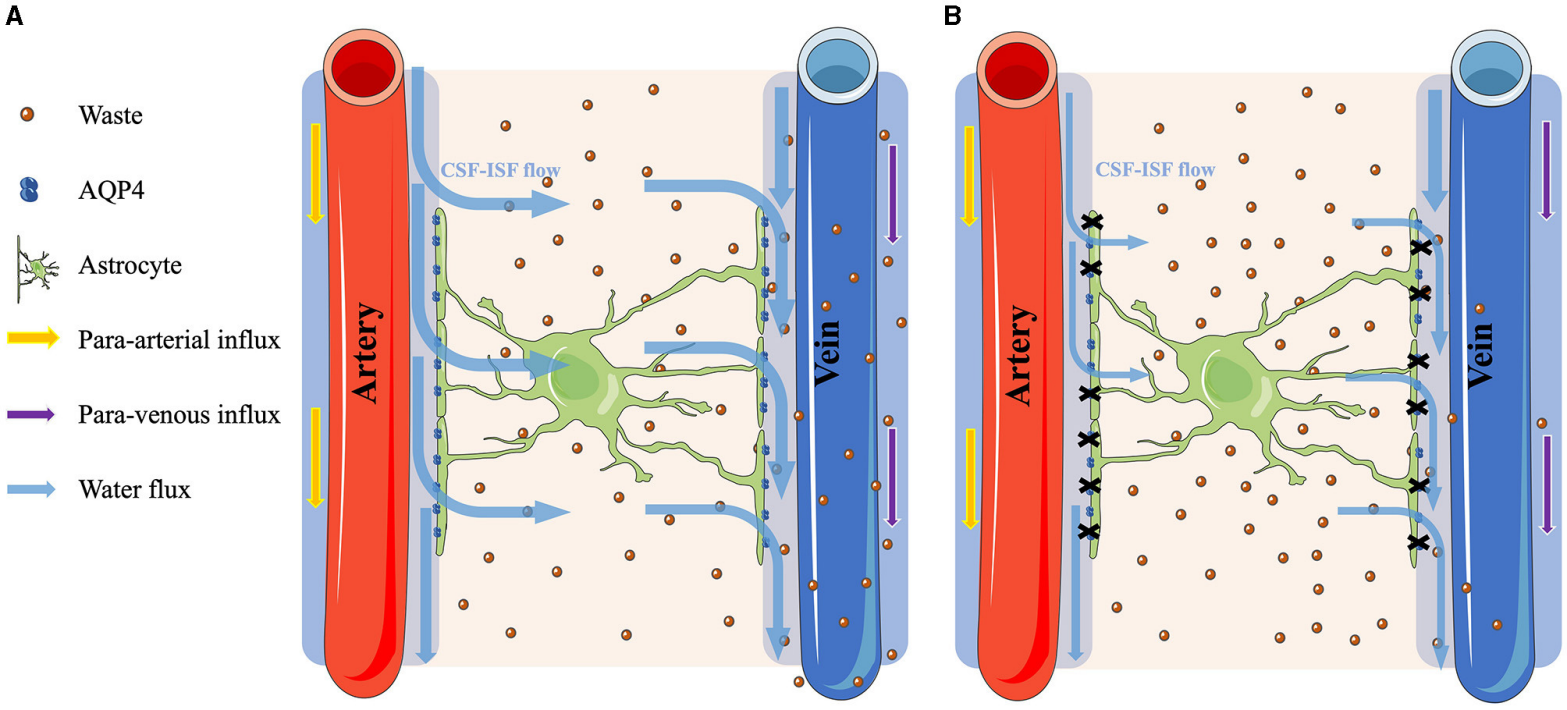
Comparison of the glymphatic system function between normal glucose metabolism and hyperglycemia conditions. **(A)** The process under the NGM condition. The cerebrospinal fluid (CSF) flows through the AQP4 water channels on the astrocytes and mixes with the interstitial fluid (ISF) to clear the brain waste. **(B)** Contrarily, under hyperglycemia conditions, the astrocytes were damaged, causing a reduction of the AQP4 water channels. Consequently, the CSF–ISF flow in the parenchyma is restricted, leading to the accumulation of metabolic waste within the brain.

In the rat study of Jiang et al. (2017) that evaluated the alteration of the glymphatic system in T2DM rats using contrast-enhanced MRI with Gd-DTPA, the clearance rate of the CSF contrast agent Gd-DTPA in T2DM rats was lower by three times as compared to that of NGM rats. Additionally, an earlier human research utilizing DTI-ALPS from dMRI to examine changes in the glymphatic system among patients with T2DM showed a notably reduced ALPS index in the T2DM group compared to the NGM group (Yang et al., 2020). Our research aligns with prior observations, suggesting a possible reduced activity of the glymphatic system in T2DM patients relative to those in the NGM group. Additionally, our results showed that patients with Pre-DM, which might be a prodromal group of patients with T2DM, also had a lower ALPS index than the participants with NGM, but there was no difference between the patients with T2DM and Pre-DM. According to the Cohen's guideline, the disparity in the ALPS index among the NGM and T2DM groups (Cohen's *d* = −1.32) was indicative of substantial effects. Notably, a similar substantial effect was observed when comparing the ALPS index among the NGM and Pre-DM groups (Cohen's *d* = −1.04). This could suggest that the glymphatic system activity in individuals with Pre-DM began to decline even before their transition to T2DM, aligning with our hypothesis. Conversely, the disparity in the ALPS index among the Pre-DM and T2DM groups (Cohen's *d* = −0.63) showed medium effects. It might mean that the operational state of the glymphatic system in individuals with Pre-DM is nearing the conditions observed in patients with T2DM. The diminished function of the glymphatic system might correlate with the observed AQP4 reductions in T2DM, given that AQP4 is crucial components of the glymphatic system responsible for waste elimination (Benveniste et al., 2019), and its reduction could compromise glymphatic system clearance efficiency. Indeed, animal studies have previously highlighted a decline in AQP4 and an increase in Aβ plaque accumulation in patients with T2DM in contrast to NGM (Zhang et al., 2016; Jiang et al., 2017; Ward et al., 2019). The hyperglycemia in T2DM could cause damage to astrocytes where AQP4 is attached and could reduce AQP4 (McMurray et al., 2016; Rehman and Akash, 2017), leading to reduced function of the glymphatic system (Figure 4).

In this investigation, the ALPS index in Pre-DM and T2DM was lower compared to NGM, the ALPS index neared the value of 1.0. Given the ALPS index approaching 1.0, denoting minimal diffusivity within the perivascular space, our findings suggest that the diffusivity along the perivascular space decreases in patients with Pre-DM and T2DM, as compared to that seen in the participants with NGM. The diffusivity within the perivascular space holds the potential to serve as an indirect indicator of the glymphatic system's functionality. For instance, the perivascular space expansion could also be a factor that reduces the clearance of GBCA with intrathecal injections caused by the stagnation of the glymphatic transport for animal studies due to the decreased diffusivity along the perivascular space (Tali et al., 2002; Öner et al., 2017; Taoka et al., 2017b; Yu et al., 2022). Patients with T2DM commonly have micro- and macro-vascular damages, including conditions like cortical atherothrombotic embolism. This heightens their susceptibility to small vessel disease and enlarged perivascular space (Giwa et al., 2012; Prasad et al., 2014). Indeed, a previous study has investigated small-penetrating cerebral arteries of pathologically diagnosed small vessel disease cases by microscopic examination of standard hematoxylin and eosin sections. The findings indicated that in conditions of T2DM, the arteriolar structure can be altered and the perivascular space in the brain might be enlarged. Such changes could have implications for the glymphatic system (Giwa et al., 2012).

There are also some reports on the vascular damage observed in patients with Pre-DM, who are at risk for developing an enlargement of the perivascular space (Nguyen et al., 2007; Roriz-Filho et al., 2009; Biessels et al., 2014; Buysschaert et al., 2015). Pre-DM can result in an overabundance of CSF, being trapped in the dilated perivascular space and pressuring the penetrating artery reducing its pulse inside the brain parenchyma (Garcia-Alloza et al., 2011). The suppression of arterial pulsation, which may serve as the glymphatic system's driving force, could negatively impact its performance (van Veluw et al., 2020). Moreover, the change in the perivascular space was associated with Aβ deposition in the brain and neuronal change (Kamagata et al., 2022; Tang et al., 2022). Recently, Kamagata et al. demonstrated not only the enlargement of the perivascular space but also the decline of the ALPS index occurring in patients with mild cognitive impairment and AD, possibly due to the impairment of the glymphatic system (Kamagata et al., 2022; Tang et al., 2022). Other reports on the glymphatic system also showed a significant relation between the ALPS index and cerebral small vessel diseases. including cerebral microbleeds, lacunes, and perivascular spaces (Ke et al., 2022; Tang et al., 2022). Thus, the cerebral neurovascular dysfunction in the Pre-DM and T2DM groups could lead to an enlargement in the perivascular space and a decrease in the ALPS index, which could cause a deterioration in the glymphatic system.

In this research, we found a notable inverse relationship between the ALPS index and HOMA-IR, which measures the level of insulin resistance. Recently, the CNS glymphatic system activity in patients with diabetes is correlated with T2DM features, such as insulin resistance (Iliff et al., 2012), which may cause atherosclerosis, leading to reduced arterial pulsation that contribute to the driving force of CSF in the glymphatic system (Jessen et al., 2015). Two studies from Korea (Lee et al., 2016) and Japan (Someya et al., 2021) found that insulin resistance evaluated by HOMA-IR and estimating clamp-derived insulin sensitivity from the oral glucose insulin sensitivity index was an independent risk factor of vascular damage, such as cortical atherothrombotic embolism and lacunar stroke, which is positively correlated with the incidence and severity of vascular damage. In a previous study involving patients without diabetes showing signs of possible myocardial ischemia, there was an inverse relationship with HOMA-IR and a low reactive hyperemic index, this suggests a strong link between insulin resistance as determined by HOMA-IR and prognostically relevant endothelial dysfunction (Westergren et al., 2016). Furthermore, Howard et al. (1996) assessed the insulin sensitivity through a venous glucose tolerance test and atherosclerosis measured by intimal–medial thickness of the carotid artery by ultrasonography. The results indicated a direct correlation between atherosclerosis and insulin resistance. Thus, the atherosclerosis caused by high insulin resistance levels may affect the ALPS index and influence the glymphatic system's activity because of the deterioration of the arterial pulsation that contributes to the driving force of CSF in the glymphatic system, thereby causing impairment glymphatic system functionality (Jessen et al., 2015; Li et al., 2021).

Moreover, the brain's insulin resistance could reduce the sensitivity of the brain to insulin and could deteriorate the glymphatic function, leading to Aβ accumulation in the neuronal insulin signal pathway with insulin resistance (Talbot, 2014). Microglial cells, upon stimulation by Aβ oligomers, initiate the activation of IRS-1 serine kinases. This activation results in phosphorylation that is markedly elevated in cerebral pyramidal cells of individual with Pre-DM and T2DM, disrupting the relay of insulin signals to subsequent targets. Such interference in signaling likely contributes to the reduction in Aβ clearance (Zhao et al., 2009; Sims-Robinson et al., 2010), which in turn could amplify the insulin resistance, as delineated in the process. Our findings demonstrated a pronounced inverse association between HOMA-IR, pertaining to insulin resistance, and the ALPS index. This association could potentially indicate the workings of the glymphatic system, further reinforcing the conclusions of previous reports. Although HOMA-IR is indicative of the sensitivity of peripheral tissues, characteristics of peripheral insulin resistance have been noted to exert an indirect influence on the CNS (Rhea et al., 2022).

First, this study acknowledges certain limitations, including the use of a small sample size from a single institution. Expanding the sample size across multiple institutions would not only bolster the statistical strength and credibility of our research but also minimize the risk of overfitting, ensuring a more comprehensive understanding of the context and constraints affecting our findings. Additionally, our investigation underscores the feasibility and potential of conducting studies with extensive samples based on the limited sample size used in this study. Second, the ROI placement for the ALPS index is currently manually. To enhance the reliability of our findings, transitioning to an objective approach, such as automated ROI placement, would be beneficial. Third, among the T2DM patients recruited in our study, 11 were on medication, which could potentially affect the ALPS index. Specifically, medications like metformin, known to alter HOMA-IR, could have influenced our results, thus introducing an additional limitation to our study. Finally, this study indirectly evaluated the glymphatic system using only the ALPS index. The glymphatic system could reportedly be also associated with the perivascular space volume based on the structural MRI findings and free water volume fraction in white matter based on the dMRI data (Kamagata et al., 2022; Tang et al., 2022). In the future, studies should use these indices and evaluate the glymphatic system by integrating the result of the analysis using these indices related to the glymphatic system.

## 5 Conclusion

In conclusion, in line with our hypothesis, there may be an early decline in the glymphatic system in individual with Pre-DM before they progress to T2DM. Furthermore, there seems to be an inverse relationship between the functionality of the glymphatic system and insulin resistance level. Through this study, we have deepened our understanding of the adverse effects of both T2DM and Pre-DM on the glymphatic system. This knowledge will help us in creating more effective preventive and therapeutic strategies to maintain brain health.

## Data availability statement

The raw data supporting the conclusions of this article will be made available by the authors, without undue reservation.

## Ethics statement

The studies involving humans were approved by Juntendo University Ethics Committee and followed ethical standards outlined in the 1964 Helsinki Declaration and its subsequent amendments or similar ethical standards. The studies were conducted in accordance with the local legislation and institutional requirements. The participants provided their written informed consent to participate in this study.

## Author contributions

RT: Conceptualization, Data curation, Formal analysis, Funding acquisition, Investigation, Methodology, Project administration, Resources, Software, Supervision, Validation, Visualization, Writing – original draft. KK: Conceptualization, Funding acquisition, Methodology, Writing – review & editing. YSa: Formal analysis, Visualization, Writing – review & editing. CA: Formal analysis, Visualization, Writing – review & editing. KT: Formal analysis, Visualization, Writing – review & editing. WU: Formal analysis, Visualization, Writing – review & editing. SY: Formal analysis, Visualization, Writing – review & editing. JK: Formal analysis, Visualization, Writing – review & editing. HT: Formal analysis, Visualization, Writing – review & editing. HN: Formal analysis, Visualization, Writing – review & editing. YSo: Conceptualization, Methodology, Supervision, Writing – review & editing. HK: Conceptualization, Methodology, Supervision, Writing – review & editing. MM: Conceptualization, Methodology, Supervision, Writing – review & editing. TA: Conceptualization, Methodology, Supervision, Writing – review & editing. AW: Conceptualization, Methodology, Supervision, Writing – review & editing. TT: Conceptualization, Methodology, Supervision, Writing – review & editing. SN: Conceptualization, Methodology, Supervision, Writing – review & editing. YT: Conceptualization, Methodology, Supervision, Writing – review & editing. HW: Conceptualization, Methodology, Supervision, Writing – review & editing. RK: Conceptualization, Methodology, Supervision, Writing – review & editing. SA: Conceptualization, Funding acquisition, Supervision, Writing – review & editing.
